# Proceedings from the 26th Annual Symposium of the International Cannabinoid Research Society: June 26–July 1, 2016

**DOI:** 10.1089/can.2017.29008.crs

**Published:** 2017-03-01

**Authors:** Mauro Maccarrone

**Affiliations:** ^1^ Department of Medicine, Campus Bio-Medico University of Rome, Rome, Italy.; ^2^ Laboratory of Lipid Neurochemistry, Santa Lucia Foundation IRCCS, Rome, Italy.

**Keywords:** central nervous system, endocannabinoid, medicinal cannabis, peripheral organs, phytocannabinoid

## Abstract

The 26^th^ Annual Symposium of the International Cannabinoid Research Society (ICRS) was held in the mountain town of Bukowina Tatrzanska (Krakow), Poland, on June 26–July 1, 2016. In this breathtaking location, newcomers and well-established investigators in the field of (endo)cannabinoid research presented exciting new data, with a good balance between preclinical and clinical topics, both in the central nervous system and in peripheral tissues. Potential indications for the therapeutic use of cannabis to treat various human disorders were also discussed in the conference. After more than a quarter of a century, ICRS proved to be once again a unique forum to share novel concepts on (endo)cannabinoid research at large, and most of the approximately 300 delegates voiced their support for having such a forum where diverse (and sometimes contrasting) ideas can be exchanged, fostering new collaborations.

In midsummer of 2016, ∼300 delegates from all over the world converged upon the Bukovina Resort, one of the most fascinating and breathtaking landmarks of Poland. Among them, ∼1/3 were PhD students and Postdoctoral fellows. All participants were driven by a strong interest toward preclinical and clinical cannabinoid and endocannabinoid research, that the International Cannabinoid Research Society (ICRS) has been able to foster as a reference forum for more than a quarter of century. The conference was a true full immersion with more than 10 h a day of lectures, poster presentations, and mutual interactions for 4 days. The symposium was coordinated by some well-established members of the Board of Directors led by the local organizer, Dr. Katarzyna Starowicz: Dr. Steve Alexander (Past President), Dr. Michelle Glass (President), Dr. Cecilia Hillard (Executive Director), Dr. Natalia Malek (Student Representative), and Dr. Jason Schechter (Managing Director). It was divided into 13 oral sessions (with a total of 84 presentations) and 2 poster sessions (with a total of 101 posters), and featured 4 special lectures: the ICRS Career Achievement Award, the ICRS Presidential Address, the Kang Tsou Memorial Lecture, and the Young Investigator Award Presentation. At the end, an ICRS business meeting and a banquet with the awards ceremony closed the conference.

For the first time, an agreement was made that the abstracts of the 26th annual symposium of ICRS would be published in *Cannabis and Cannabinoid Research*, and the readers are encouraged to look at them to appreciate the wealth of data that were presented and shared among the delegates. In this article, the general structure of the meeting will be summarized, as a compass to navigate the contents of the symposium.

## Day 1

The first day included five oral sessions on the following subjects: (1) Medicinal *Cannabis* I, that featured six presentations and was chaired by Dr. Dror Robinson (HaSharon Hospital of Petah Tikwa, Israel) and Dr. Mark A. Ware (McGill University of Montreal, Canada); the latter also delivered the inaugural talk on “Pharmacovigilance of medical cannabis: preliminary results from the Quebec cannabis registry,” whereas the remaining five lectures were delivered by D. Robinson, P. Lucas, L. Bar-Lev Schleider, L. Saynor, and M.A. Kowal; (2) Endocannabinoid Transport/Dynamics, that featured five lectures (by J. Bialecki, M.W. Elmes, M. Kaczocha, Y. Chen, and C.J. Hillard) and was chaired by Dr. Martin Kaczocha (New York University at Stony Brook, NY) and Dr. Matt Hill (University of Calgary, Canada); (3) Cardiovascular System, that featured five lectures (by E. Brailoiu, B. Malinowska, C.T. Choy, R. Mohanraj, and E.E. McNaughton) and was chaired by Dr. Saoirse O'Sullivan (University of Nottingham, United Kingdom) and Dr. Wing Sze Vanessa Ho (St. George's University of London, United Kingdom); (4) Behavioral Neuroscience I–Pain and Sleep, that featured six lectures (by X. Sun, D.J. Morgan, J. Wilkerson, A.M. Myers, C.R. McCurdy, and L.M. Carey) and was chaired by Dr. Aron Lichtman (Virginia Commonwealth University at Henrico, VA) and Dr. Sara Jane Ward (Temple University at Philadelphia, PA); (5) Focus on CBD, that featured eight lectures (by R. Tuma, J.T. Toguri, E. Kozela, D.R. Smith, M. Ceprian, T. Bakas, D. Couch, and A. Ligresti) and was chaired by Dr. John Ashton (University of Otago, New Zealand) and Dr. Alessia Ligresti (Institute of Biomolecular Chemistry at Pozzuoli, Italy).

Day 1 also hosted the ICRS Career Achievement Award lecture, delivered between oral sessions 1 and 2 by the award winner Dr. Dale Deutsch (New York University at Stony Brook, NY) on “Anandamide uptake, transport and inactivation: studies bridging two centuries.” This major event was chaired by Dr. Allyn Howlett (Wake Forest School of Medicine at Winston-Salem, NC), and recognized a life-long contribution of one of the best known scientists in the field. In addition, a NIDA Lunch & Mentoring event took place between oral sessions 2 and 3, and was received as a useful guidance on NIDA funding policy.

## Day 2

The second day of the conference included three oral sessions and kicked off with the one on Focus on CB_2_. This session featured eight presentations (by F. Espejo-Porras, A. Lopez, Z.-X. Xi, W.J. Redmond, A.-C. Schmole, N. Malek, D. Kho, and Z.V. Varga) and was chaired by Dr. Scott Graham (University of Auckland, New Zealand) and Dr. Katarzyna Starowicz (Polish Academy of Sciences at Krakow, Poland). The second oral session was chaired by Dr. Natasha Grimsey (University of Auckland, New Zealand) and Dr. Sharon Anavi-Goffer (Ariel University, Israel) and covered the topic of Receptors, Probes and Signaling with eight presentations (delivered by M. Soethoudt, U. Grether, K. Eldeeb, B. Szabo, T.W. Grim, S. Anavi-Goffer, M.A. Lingerfelt, and B. Thomas). Then, the last oral session, chaired by Dr. Andras Bilkei-Gorzo (Universitatsklinikum Bonn, Germany) and Dr. Jose Martinez-Orgado (Hospital Clinico San Carlo of Madrid, Spain) addressed Neuroprotection/Neuroinflammation/Neurogenesis with seven presentations (by A. Piyanova, T. Kino, K.S. De Coteau, B. Ignatowska-Jankowska, L. Barata, P. Morales, and Y. Sarne).

In addition, Poster Session I was held in the afternoon of day 2, and covered 8 topics with 53 posters: 12 addressed Peripheral Inflammation and Immune System; 5 Behavioral Neuroscience I; 4 Cardiovascular; 6 CB1 Receptors–Signaling, Novel Probes, Etc.; 4 Cannabis; 5 Cancer; 8 Receptors–Signaling, Novel Probes, etc.; 9 Endocannabinoids–Synthesis and Metabolism.

After the oral sessions, day 2 also featured the ICRS Presidential Address delivered by Dr. Andrew Tobin (University of Leicester, United Kingdom) on “Moving from mechanisms of GPCR functionality to new drugs.” This major event for ICRS was chaired by the ICRS President Dr. Michelle Glass (University of Auckland, New Zealand).

## Day 3

Two more oral sessions were held on the third day of the conference: (1) Metabolism, Endocrine, Diabetes, that featured seven presentations (delivered by T. Jourdan, L. Hinden, D.I. Brierley, M. Maccarrone, I. Knani, Y. Wang, and S.E.M. de Bruijn) and was chaired by Dr. Claire Williams (University of Reading, United Kingdom) and Dr. Pal Pacher (National Institutes of Health at Rockville, MD); (2) Medicinal *Cannabis* II, that featured six lectures (by J.M. McPartland, A. Hazenkamp, M. Haney, G. Jager, D. Hudson, and E. Russo) and was chaired by Dr. Ethan Russo (Phytecs at Vashon, WA) and Dr. John McPartland (GW Pharma at Middlebury, VT).

After the oral sessions, day 3 also featured the Kang Tsou Memorial Lecture, delivered by Dr. Andrej Dziembowski (Warsaw University, Poland) on “Human exosome complex in health and disease.” This traditional ICRS lecture was chaired by the local organizer Dr. Katarzyna Starowicz (Polish Academy of Sciences at Krakow, Poland).

After 2.5 days of marathon for the brain, delegates had the chance to rest their minds and move their bodies. Indeed, the whole afternoon of day 3 was free for a very typical excursion in the Chocholowska valley of the Tatra national park, either in “hiking mode” (for those who were better fit) or in “horse carriage mode” (for the others). This outing was really spectacular, and again a nice opportunity to have lovely scientific and nonscientific discussions among all delegates and for the new comers to walk and share ideas with those more established in the field.

## Day 4

The last day of the conference included three oral sessions on the following subjects: (1) Addiction, that featured four presentations (by J. Orihuel, E.L. Gardner, J. Lovelace, and J.E. Schlosburg), and was chaired by Dr. Joel Schlosburg (The Scripps Research Institute at La Jolla, CA) and Dr. Eliot Gardner (National Institue on Drug Abuse at Baltimore, MD); (2) Behavioral Neuroscience II–Anxiety, Stress, that featured five presentations (delivered by K.R. Trexler, V. Micale, H.A. Vecchiarelli, N. Gu, and R.J. Bluett), and was chaired by Dr. Steven Kinsey (West Virginia University at Morgantown, WV) and Dr. Sachin Patel (Vanderbilt University Medical Center at Nashville, TN); (3) Endocannabinoid Metabolism, that featured nine lectures (by M.P. Baggelaar, M. Garle, M.S. Crowe, E. Leishman, A. Olah, H. Deng, J.J. McDougall, K.L. Wills, and A. Howlett) and was chaired by Dr. Heather Bradshaw (Indiana University at Bloomington, IN) and Dr. Mario van der Stelt (Leyden University, The Netherlands). The closing lecture on “Endocannabinoid system receptors and enzymes are expressed in early developing human brain organoids” was delivered by Dr. Allyn Howlett (Wake Forest School of Medicine at Winston-Salem, NC), one of the founders of the field who explored an entirely new territory of research in 3D cellular systems.

In addition, Poster Session II was held in the afternoon of day 4, and covered 6 topics with 48 posters: 12 addressed Behavioral Neuroscience II–Pain, Anxiety, and Fear; 4 Visual System; 5 GPR55; 9 Neuroinflammation, Neuroprotection, Neuropsychiatric Disorders, Cognition; 8 Medicinal Cannabis; 10 Endocrine, Gastrointestinal, Diabetes.

Day 4 also hosted the Young Investigator Award Presentation, delivered between oral sessions 1 and 2 by the award winner Dr. Sachin Patel (Vanderbilt University Medical Center at Nashville, TN) on “Endocannabinoids as a homeostatic signal counteracting adverse effects of stress.” This major event for ICRS community was chaired by Dr. Cecilia Hillard (Medical College of Wisconsin at Milwakee, WI), and recognized one of the rising stars in the ICRS community.

At the end of the scientific sessions a well-attended and alive ICRS business meeting took place, where it was reported that the society has reached the remarkable number of 515 regular members. Then, there was the traditional Awards Ceremony during the ICRS banquet, where the following Predoctoral Prize winners were announced: Rebecca Bluett (Vanderbilt University), who received the Billy Martin Prize, David Finlay (University of Auckland), Hui Deng (Leiden University), Natalia Malek (Polish Academy of Sciences at Krakow), Molly Crowe (West Virginia University), and Christina Miyabe (Northeastern University). The Postdoctoral Prize winners were as follows: Dr. Luigi Bellocchio (INSERM Bordeaux), who received the J. Michael Walker Prize, Dr. William John Redmond (Université de Montréal), Dr. Erin Rock (University of Guelph), Dr. Anne Schmöle (University of Bonn), and Dr. Erica Zamberletti (University of Insubria). During the banquet also two major ICRS awards were presented: the Young Investigator Award to Dr. Saoirse O'Sullivan (University of Nottingham), and the ICRS Career Achievement Award to Dr. Linda Parker (University of Guelph). Finally, the most important recognition that ICRS can deliver, that is, the Mechoulam Award, was presented to Dr. Mauro Maccarrone (Campus Bio-Medico University of Rome) “for outstanding contribution to cannabinoid research.” To make this conclusive event even more memorable, Dr. Raphael Mechoulam himself presented the award to the winner, who was the 18th scientists to receive it in ICRS history.

## Concluding Remarks

The ICRS 2016 symposium offered once again state-of-the-art presentations, with a very good balance between preclinical and clinical studies on central and peripheral aspects of (endo)cannabinoid signaling. Very active discussions after each talk or at the posters, which stayed on display long enough to facilitate cross-talks, further confirmed that the field is alive and kicking. The merging of basic scientists and clinicians was unanimously recognized as a key strength for gaining a better understanding of the potential therapeutic exploitation of (endo)cannabinoids in several human diseases. The high quality and varied perspectives offered by the speakers—as well as the richness of the discussions—underscored the maturity of the (endo)cannabinoid research field and its readiness to take on the medical and societal challenges lying ahead.

Against this background, all delegates marked their agenda for the 27th Annual Symposium, to be held in Montreal (Canada) between 22 and 27 June 2017.

## Abbreviation Used

ICRS: International Cannabinoid Research Society

